# An Effective, Economical and Ultra-Fast Method for Hydrophobic Modification of NCC Using Poly(Methylhydrogen)Siloxane

**DOI:** 10.3390/polym11060963

**Published:** 2019-06-03

**Authors:** Xueqing You, Qingjian Hu, Xiaoyong Hu, Hanxian Chen, Wenbin Yang, Xinxiang Zhang

**Affiliations:** College of Material Engineering, Fujian Agriculture and Forestry University, Fuzhou 350002, China; 15880884064@163.com (X.Y.); qjhu0215@163.com (Q.H.); hxy1198578262@163.com (X.H.); hanxian1229@163.com (H.C.); fafuywb@163.com (W.Y.)

**Keywords:** nanocellulose, hydrophobic, surface modification, poly(methylhydrogen)siloxane, thermal stability

## Abstract

Poor compatibility between nanocellulose crystals (NCCs) and major polymers has limited the application of NCC as bio-reinforcements. In this work, an effective and ultra-fast method was investigated to significantly improve the hydrophobicity of NCC by using poly(methylhydrogen)siloxane (PMHS) as modifier. PMHS possessed amounts of reactive –Si–H groups and hydrophobic –CH_3_ groups. The former groups were reactive with the hydroxyl groups of NCC, while the latter groups afforded NCC very low surface energy. As the weight ratio of PMHS to NCC was only 0.0005%, the hydrophobicity of NCC was significantly improved by increasing the water contact angle of NCC from 0° to 134°. The effect of weight ratio of PMHS to NCC and the hydrogen content of –Si–H in PMHS on the hydrophobicity and thermal stability was investigated in detail by Fourier transform infrared spectroscopy (FTIR), (X-ray Diffraction) XRD and (thermogravimetric analysis) TGA. The results indicated that PMHS chains were covalently grafted onto NCC and PMHS modification improved the thermal stability of NCC.

## 1. Introduction

Nanocellulose crystals (NCCs), extracted from hierarchical structure of cellulose, demonstrate a number of advantages, such as exceptional mechanical properties, high aspect ratio, low density and availability [1,2,3,4]. Hence, they have been widely used as the reinforcements in bionanocomposites. However, their application is limited by poor compatibility between NCC and nonpolar polymer media or matrices due to polar properties of NCC [5]. Chemical modification of NCC was proposed to address this issue [6,7,8,9,10,11,12,13,14,15,16]. The methods for chemical modification of NCC include isocyanation [6], esterification [6], silanation [7,8,9,10], oxidation [11,12], and acylation [13,14,15]. Siqueira et al. [5] prepared hydrophobic NCC by using n-octadel isocyanate (C_18_H_37_NCO) as modifier. Lin et al. [10] realized very hydrophobic NCC by a two-step silanation method by using KH560 (γ-(2,3-epoxyproxy) propytrimethoxysilane) as a linker and dodecyltrimethoxysilane (DTMOS) as hydrophobic modifier. Menezes et al. [15] modified NCC with organic fatty acid chlorides with different aliphatic chain length by using hexanoyl chloride, lauroyl chloride and stearoyl chloride as modifiers. These methods significantly improved the hydrophobicity of NCC. However, a complicated process was required, and the modifiers were quite expensive.

Among these modification methods, silanation modification of NCC has been gaining more and more attention [10,16,17]. Recently, NCC was reported to be modified by silylation using 3-aminopropyltriethoxysilane (APTES) [16] and 3-2-(2-aminoethylamino)ethylamino propyl-trimethoxysilane (TAMS) [17]. However, the hydrophobicity of modified NCC by APTES and TAMS should be improved. In this work, we describe an effective, economical and ultra-fast approach for hydrophobic modification of NCC by using PMHS as modifier. PMHS chains had many Si–H groups and –Si–CH_3_ groups. The Si-H groups possessed ultra-high reactivity to hydroxyl groups of NCC in the presence of Karstedt catalyst and then the PMHS chains with low surface energy would be covalently grafted onto the surface of NCC. In addition, PMHS are mass-produced products in silicone industry and therefore are easily handled and inexpensive. Finally, the effect of weight ratio of PMHS to NCC and hydrogen content of –Si–H in PMHS on hydrophobicity and thermal stability of NCC was investigated in detail by FTIR (Fourier transform infrared spectroscopy), XRD (X-ray diffraction) and TGA (thermo gravimetric analyzer).

## 2. Materials and Methods

### 2.1. Materials

Microcrystalline cellulose (MCC) with particle size of 50 μm was purchased from Huzhou Yinhuxinwang Chemical Co., Ltd., Huzhou, China. H_2_SO_4_ and hexane were of analytical grade and supplied by Sinpharm Chemical Reagent Co., Ltd., Shanghai, China. PMHS and Kastredt catalyst (platinum-1,3-divinyl-1,1,3,3-tetramethyldisiloxane) were supplied by Chenguang Research Institute of Chemical Industry, Chengdu, China.

### 2.2. Preparation of NCC

NCC was fabricated by Tang’s method which included H_2_SO_4_ hydrolysis of MCC assisted with ultrasonic treatment [18]. H_2_SO_4_ hydrolysis was performed at 52 °C with 65 wt% H_2_SO_4_ for 2 h under mechanical stirring. Then, the suspension was washed until neutrality by successive centrifugations at the relative centrifugal relative force (CRF) of 10,833 g and dialyzed against distilled water for 5 days. Finally, the NCC powders were obtained after freeze-drying.

### 2.3. Hydrophobic Modification of NCC

PMHS was used as hydrophobic surface modifier. The hydrogen contents of –Si–H in PMHS was 0.18%, 1.0% and 1.5%, and the resultant PMHS was named as 0.18%PMHS, 1.0%PMHS and 1.5%PMHS, respectively. The modifier solution was prepared by mixing x g PMHS, (50-x) g hexane and 2 droplets of Kastredt catalyst (platinum-1,3-divinyl-1,1,3,3-tetramethyldisiloxane) together. Then, 0.5 g of NCC powder was added to the modifier solution at room temperature for 10 min under vigorous mechanical stirring. The weight ratio of 0.18%PMHS to NCC in modifier solution was 5%, 10%, 20%, 50%, 80%, 100% and 150%, while that of 1.0%PMHS and 1.5%PMHS was both 0.0005%, 0.001%, 0.005%, 0.01%, 0.05%, 0.2% and 0.5%. After modification, the modified NCC were filtered with 0.22 μm PVDF (polyvinylidene fluoride) filters and washed with hexane for three times.

### 2.4. Characterization

WCA (water contact angle) measurement was executed using the sessile drop configuration at room temperature on Krüss DSA-30 instrument (Hamburg, Germany) equipped with a CDD (Charge Coupled Device) camera. For WCA measurement, NCC powder was spread onto the surface of double-side tap. The volume of water droplets for all measurements was set at 5 μL. The dynamic contact along with time was recorded. The FTIR spectra of all samples were recorded between 400 and 4000 cm^−1^ using a Bruker Tensor 27 FTIR spectrophotometer. NCC, 0.18%PMHS, 1.0%PMHS and 1.5%PMHS modified NCC samples were respectively mixed with analytical grade KBr powders and then pressed into pellet. The total of 32 scans was obtained, using a resolution of 4 cm^−1^. The crystalline structure and crystallinity index (CrI) changes of the samples were tested by X-ray diffractometer (Rigaku D/Max-Ra, Rigaku, Toyko, Japan) analysis using Cu Kα radiation. Test were taken from 2θ = 6° to 60° at a step size of 0.1°. The CrI was calculated according to Segal’s method [19]. The equation is an empirical method for evaluating the degree of crystallinity in the NCC.

CrI(%) = (I_002_ − I_am_)/I_002_·100 (1)

where I_002_ is the maximum intensity of the (002) lattice diffraction peak at 2θ ≈ 22° and I_am_ is the lowest intensity at 2θ ≈ 18°, representing the amorphous part of the sample. The thermal stability of the samples was investigated by TGA (Netasch, TG209 F1, Selb, Germany). The amount of sample taken for each test was approximately 5 mg. All tests were executed under a nitrogen atmosphere with a gas flow 10 mL/min and heated from 30 °C to 600 °C at a heating rate of 10 °C/min.

## 3. Results and Discussion

### 3.1. Hydrophobicity of NCC

**Figure 1:**
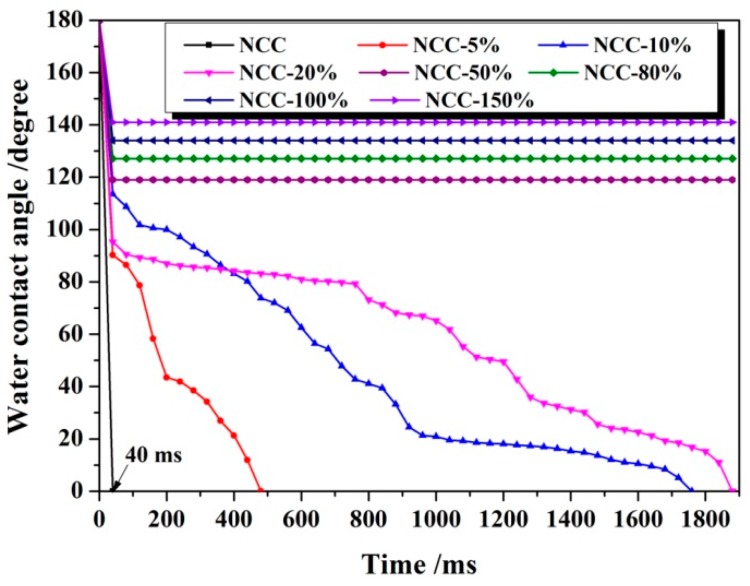
Dynamic contact angle of 0.18%PMHS modified NCC along with time.

**Figure 2:**
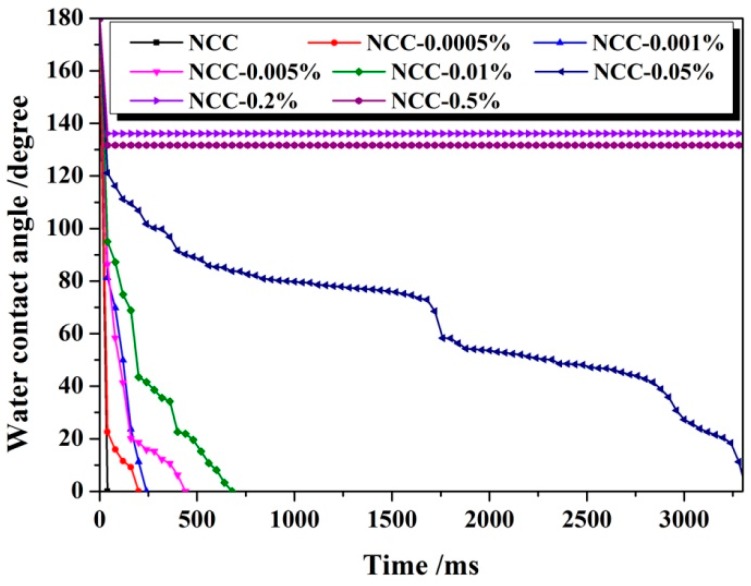
Dynamic contact angle of 1.0%PMHS modified NCC along with time.

**Figure 3:**
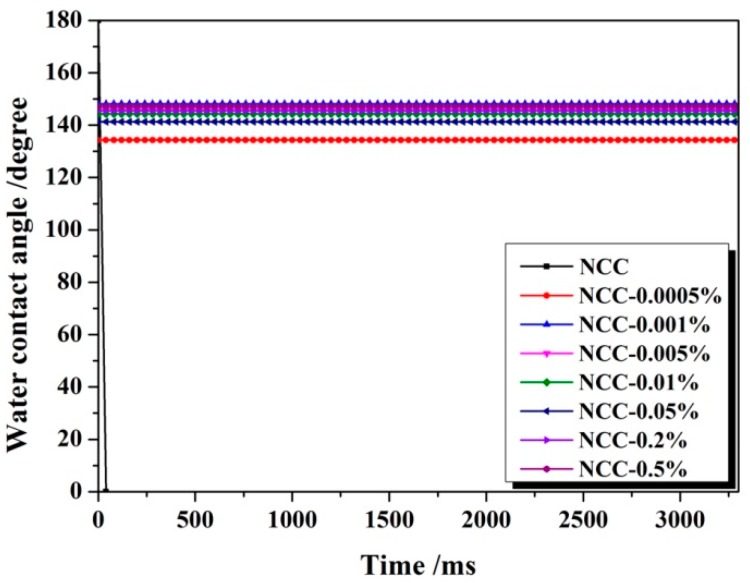
Dynamic contact angle of 1.5%PMHS modified NCC along with time.

The dynamic contact angle along with time was recorded and showed in Figure 1, Figure 2 and Figure 3. Figure 1 showed the change in water contact angle of 0.18%PMHS modified NCC as a function of test time. For unmodified NCC, the water drop was adsorbed by NCC immediately within 40 ms. Increasing the weight ratio of PMHS to NCC, the hydrophobicity of NCC was significantly improved. As the weight ratio of 0.18%PMHS to NCC lowered than 50%, the water droplets would also adsorbed by the modified NCC. However, the adsorption process was obviously inhibited by 0.18%PMHS modification. The time for total adsorption of water droplets was significantly increased from 40 ms to 1900 ms. As the weight ratio of 0.18%PMHS to NCC increased to 50%, 80%, 100% and 150%, the modified NCC could obtain a stable water droplet, and the static contact angle was recorded to be 119°, 127°, 134°, 141°, respectively.

Figure 2 showed the effect of weight ratio of 1.0%PMHS to NCC on the hydrophobicity of NCC. Compared with 0.18%PMHS, 1.0%PMHS was more effective in hydrophobic modification of NCC. As the weight ratio of 1.0%PMHS to NCC was only 0.05%, the modified NCC presented a stable water droplet on its surface, and the static water contact angle was 136°.

Figure 3 revealed the effect of weight ratio of 1.5% PMHS to NCC on the hydrophobicity of NCC. As showed in Figure 3, 1.5%PMHS was an excellent hydrophobic modifier for NCC. As the weight ratio of 1.5%PMHS to NCC was only 0.0005%, the modified NCC was totally hydrophobic with a water contact angle of 134°. As the weight ratio of 1.5%PMHS to NCC was increased further to 0.5%, the water contact angle was gradually increased to 147°. Finally, it could be concluded that 1.5%PMHS was the best candidate for hydrophobic modification of NCC, and it can be inferred that the modification efficiency of NCC by PMHS increased with hydrogen content of –Si-H in PMHS.

### 3.2. FTIR Characterization

**Figure 4:**
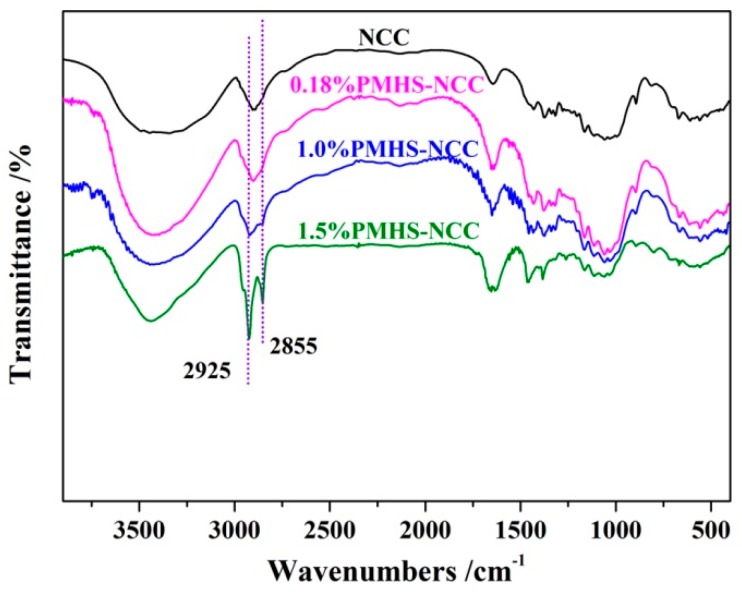
The FTIR spectra of unmodified NCC and NCC modified by 0.18%PMHS, 1.0%PMHS and 1.5%PMHS.

According to the chemical structure of PMHS, it could be deduced that, after PMHS modification, there would be lots of hydrophobic –Si–CH_3_ groups grafted onto the surface of NCC and corresponding absorption bands in FTIR spectra. Figure 4 showed the change in chemical composition of NCC before and after PMHS modification. After PMHS modification, the modified NCC had been washed by hexane for three times to remove PMHS which was not grafted onto NCC. Compared to the unmodified NCC, the PMHS modified NCC showed additional absorption band at 2855 cm^−1^. Absorption band at 2855 cm^−1^ was assigned to the –Si–CH_3_ groups [20]. In addition, during modification process, lots of bubbles were observed due to the dehydrogenation between –Si–H of PMHS and –OH of NCC. Therefore, it can be concluded that the PMHS chains were covalently grafted onto the surface of NCC, which improved significantly the hydrophobicity of NCC.

Figure 4 also revealed that the intensity of absorption bands at 2855 cm^−1^ increased with increasing hydrogen content of –Si–H in PMHS. This was in good agreement with results of water contact angle, in which 1.5%PMHS was demonstrated to be the best modifier for NCC.

### 3.3. Difference in PMHS Structure

**Figure 5:**
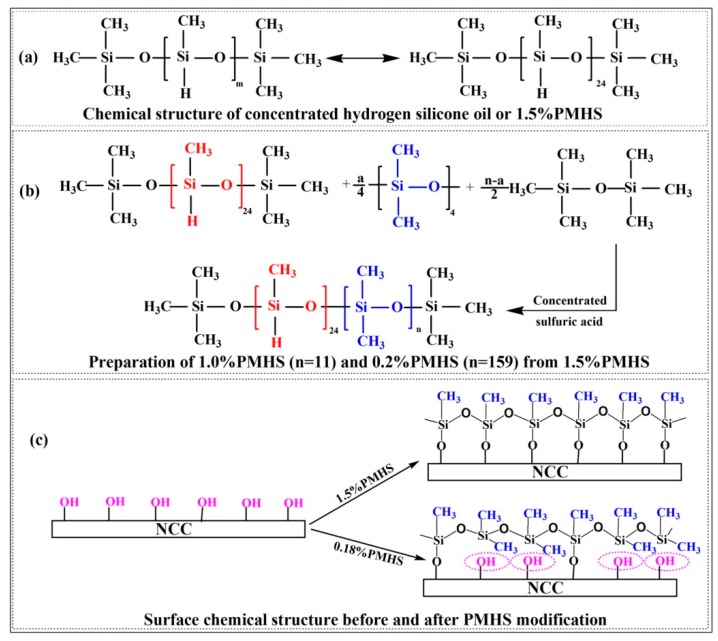
Chemical structure of 1.5%PMHS (**a**); Preparation of 1.0%PMHS and 0.2%PMHS from 1.5%PMHS (**b**); Surface chemical structure before and after PMHS modification (**c**).

As discussed before, the modification efficiency of NCC with PMHS was closely related to hydrogen content of –Si–H in PMHS. PMHS is a dimethyl polysiloxane with the general structure of (CH_3_)_3_SiO(CH_3_HSiO)*_m_*((CH_3_)_2_SiO)*_n_*Si(CH_3_)_3_ [21]. There are two kinds of repeat units on the PMHS molecule, which contains abundant reactive Si-H groups and hydrophobic –CH_3_ groups. Figure 5a is the chemical structure of 1.5%PMHS. 1.5%PMHS is the concentrated hydrogen silicone oil, which has no –Si(CH_3_)_2_O– repeat unit. The number of repeat unit “m” can be calculated to be 24. As shown in Figure 5b, 1.0%PMHS and 0.2%PMHS were derived from the concentrated hydrogen silicone oil (1.5%PMHS), and the number of repeat units of –(CH_3_)_2_SiO– could be calculated to be 11 and 159, respectively. Without regard to the bond angle of –Si–O-Si– and –Si–O–C–, the theoretical extended length of 0.18%PMHS, 1.0%PMHS and 1.5%PMHS is estimated to 60.7 nm, 12.2 nm and 8.6 nm, respectively (the bond length of Si–O and Si–C is 0.164 nm and 0.186 nm, respectively). The most striking difference of three kinds of PMHS was the chain lengths and the hydrogen content of –Si-H. The chain length of 0.18%PMHS was 7 times longer than that of 1.5%PMHS.

As shown in Figure 5c, before PMHS modification, the surface of NCC was abundant in hydroxyl groups, resulting in a very hydrophilic property of NCC. After PMHS modification, PMHS chains with amounts of hydrophobic methyl groups were grafted onto the surface of NCC. This was in good agreement with results from water contact angle characterization. In addition, as shown in Figure 5c, PMHS with different chemical structure had different modification efficiency on NCC. The chemical structure of 1.5%PMHS revealed that PMHS chain was only consisted of –CH_3_HSiO– units. In addition, 1.5%PMHS chain was short, and therefore the 1.5%PMHS chains could react more effectively with the –OH groups on NCC surface. Therefore, as showed in Figure 5c, more –OH groups on surface of NCC were replaced by PMHS chains. However, for 0.18%PMHS, it was much longer and had amounts of inert –CH_3_HSiO– units. As showed in Figure 5c, due to the steric hindrance of inert -Si(CH_3_)_2_O- units and long PMHS chain length, the dehydrogenation between 0.18%PMHS and NCC was hindered, and hence, the modification efficiency was worse than that of 1.5%PMHS.

### 3.4. Crystallinity of NCC

**Figure 6:**
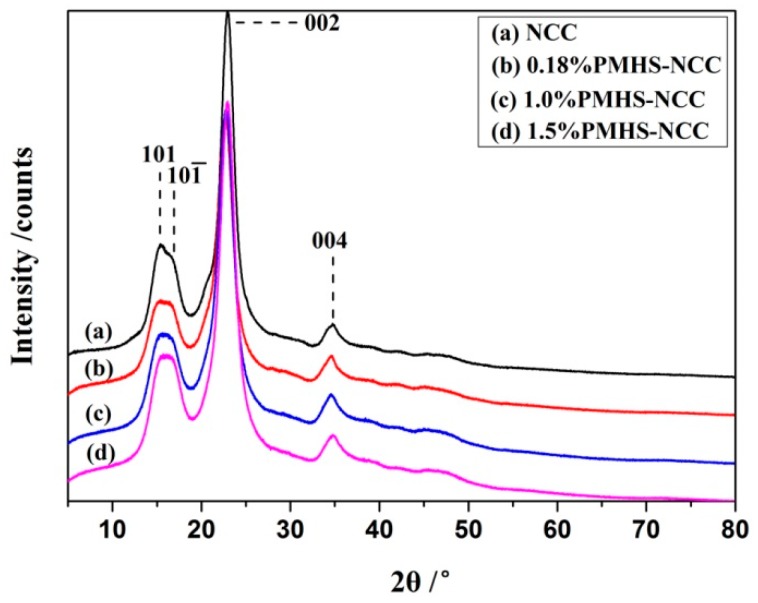
XRD patterns of NCC before and after PMHS modification.

Chemical modification performed on NCC could affect the crystallinity of cellulose [22]. To investigate the effect of PMHS modification on crystalline properties of NCC, the crystallinity index and crystalline dimensions in different planes of NCC were estimated by X-ray diffraction analysis. As showed in Figure 6, all samples had similar diffraction patterns with four diffraction peaks at 2θ = 15.2°, 16.2°, 22.8° and 34.7°, corresponding to the diffraction planes of 101, 10$\bar{1}$, 002 and 004 crystallographic planes, respectively, which were in agreement with the characteristic diffraction peaks of cellulose I [15,23]. This indicated that the crystalline type of cellulose is not altered after PMHS modification. No peak shifting or appearance of new peaks was observed. This is further evidence to suggest that the surface modification of NCC with PMHS occurred essentially only on the surface of NCC [24]. The crystallinity of NCC before and after PMHS modification as determined using the Segal-equation is presented in Table 1. It could be seen that the PMHS modification led to a slight decrease in the NCC crystallinity.

Crystalline dimension for (110), (101), and (200) planes of NCC before and after PMHS modification can be obtained from XRD measurement, and data are showed in Table 1. According to prior report [23], the number of hydroxyl groups per unit surface area (*N*_0_) on NCC can be calculated according to follow equation.

(2) $$N_{0}=\frac{120\left(\mathrm{OH}\right)}{N_{A}\left(2A_{101}+2A_{10\bar{1}}\right)}$$

*N_A_* = 6.02·10^23^ (Avogadro’s number) (3)

It is observed that the values for *N*_0_ decreased with increasing hydrogen content of –Si-H in PMHS. This result is in good agreement with the results discussed earlier.

### 3.5. Thermal Stability of NCC

**Figure 7:**
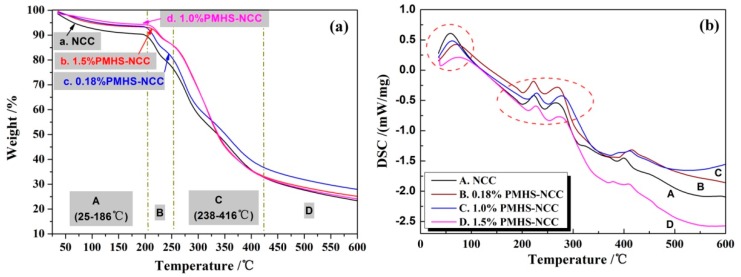
TG curves (**a**) and DSC curves (**b**) for unmodified and modified NCC.

Thermal stability is also a vital factor, especially when NCC is worked as reinforcement for polymers because elevated temperatures is a necessary for melt processing [22]. The crystallinity of NCC is high due to the hydrolysis of amorphous region of cellulose, and therefore the thermal stability of NCC is good while compared with many other biomaterials. However, as discussed before, PMHS modification slightly decreased the crystallinity of NCC, and the effect of PMHS modification on thermal stability of NCC should be carried out. Figure 7a showed the thermal degradation behavior of NCC before and after PMHS modification. All sample displayed a weight loss from room temperature to 186 °C. It was ascribed to the evaporation of absorbed water because of the hydrophilic character of the NCC surface [15]. This effect was decreased significantly for the sample with PMHS modification. It was obviously ascribed to a lower accessibility of NCC surface to water after the PMHS modification [22]. In addition, as the hydrogen content of –Si–H in PMHS increased from 0.18% to 1.5%, the weight loss of physically absorbed water decreased obviously from 6.14% to 4.17%. This is in good agreement with results from FTIR and WCA characterization, in which more hydrophobic –CH_3_ groups and better hydrophobicity were observed for 1.5%PMHDS modified NCC. Thermal decomposition of all spectra began at temperature around 186 °C. The weight loss of stage B between 186–238 °C. corresponded to glucose dehydration. Then a higher weight loss was observed in the range 238–416 °C. (stage C). The weight loss results were listed in Table 1. A comparison of PMHS modified NCC to unmodified NCC showed increased weight loss between 238 °C and 416 °C, indicating the decomposition of methyl groups for PMHS chains. In Figure 7b, it could be seen that the corresponding endothermic peak of DSC curve at around 250 °C. These results implied that the PMHS modification had little damage on the thermal stability of NCC.

## 4. Conclusions

An effective, economical and ultra-fast method was investigated to modify nanocellulose crystal (NCC) using poly(methylhydrogen)siloxane (PMHS) as modifier. PMHS modification significantly improved the hydrophobicity of NCC by increasing its water contact angle from 0 to about 140. The hydrophobic modification efficiency of NCC is proportional to the hydrogen content of –Si-H in PMHS. PMHS chains were covalently grafted onto the surface of NCC by dehydrogenation between –Si-H of PMHS and –OH of NCC. After PMHS modification, the crystallinity of NCC slightly decreased, while the thermal stability was almost unaffected.

## Figures and Tables

**Table 1:** Degree of crystallinity, crystalline dimensions, the number of hydroxyl groups per unit surface area (N_0_) and weight lose between 238–146 °C for unmodified and modified NCC.

Sample	χ_c_	Crystalline Dimensions (nm)			N_0_·10^−3^ (mmol·m^−2^)	Weight/% (238–416 °C)
		{101}	{10ī}	{002}		
NCC	78.51%	6.7	8.3	8.0	6.656	46.35
0.18%PMHS-NCC	76.59%	7.0	8.4	8.3	6.472	46.24
1.0%PMHS-NCC	74.90%	7.5	8.0	8.2	6.430	53.97
1.5%PMHS-NCC	73.71%	7.4	11.5	8.2	5.273	54.46
